# Catechin relieves hypoxia/reoxygenation‐induced myocardial cell apoptosis via down‐regulating lncRNA MIAT

**DOI:** 10.1111/jcmm.14919

**Published:** 2020-01-19

**Authors:** Lin Cong, Yisheng Su, Dazhen Wei, Lu Qian, Dawei Xing, Jialin Pan, Ye Chen, Mingyuan Huang

**Affiliations:** ^1^ Department of Cardiology The Second Affiliated Hospital and Yuying Children's Hospital of Wenzhou Medical University Wenzhou China; ^2^ Department of Cardiology Cardiovascular Key Laboratory of Zhejiang Province The Second Affiliated Hospital Zhejiang University School of Medicine Hangzhou China; ^3^ Department of Intensive Care Unit The Second Affiliated Hospital and Yuying Children's Hospital of Wenzhou Medical University Wenzhou China

**Keywords:** Akt/Gsk‐3β, catechin, hypoxia/reoxygenation, lncRNA MIAT, myocardial cell apoptosis

## Abstract

**Background:**

Catechin protects heart from myocardial ischaemia/reperfusion (MI/R) injury. However, whether catechin inhibits H/R‐induced myocardial cell apoptosis is largely unknown.

**Objective:**

This study aims to investigate the underlying mechanism of catechin in inhibiting the apoptosis of H/R‐induced myocardial cells.

**Methods:**

LncRNA MIAT expression was detected by qRT‐PCR. Cell viability of H9C2 cells was detected using CCK‐8 assay. The apoptosis of H9C2 cells was detected by flow cytometry. The interaction between CREB and MIAT promoter regions was confirmed by dual‐luciferase reporter gene assay and ChIP assay.

**Results:**

In MI/R rats, catechin improved heart function and down‐regulated lncRNA MIAT expression in myocardial tissue. In H/R‐induced H9C2 cells, catechin protected against cell apoptosis, and lncRNA MIAT overexpression attenuated this protective effect of catechin. We confirmed that transcription factor CREB could bind to MIAT promoter region, and catechin suppressed lncRNA MIAT expression through up‐regulating CREB. Catechin improved mitochondrial function and relieved apoptosis through promoting Akt/Gsk‐3β activation. In addition, MIAT inhibited Akt/Gsk‐3β activation and promoted cell apoptosis in H/R‐induced H9C2 cells. Finally, we found catechin promoted Akt/Gsk‐3β activation through inhibiting MIAT expression in H/R‐induced H9C2 cells.

**Conclusion:**

Catechin relieved H/R‐induced myocardial cell apoptosis through regulating CREB/lncRNA MIAT/Akt/Gsk‐3β pathway.

## 1 INTRODUCTION

Myocardial infarction is a leading cause of death in China that can be caused by many factors, such as the thrombotic occlusion of coronary vessels, coronary artery atherosclerosis, macrovascular and/or microvascular pathologic changes, resulting in obstructed arterial inflow (ischaemia) to heart and the damage of myocardium, which finally leads to cardiac failure and sudden cardiac death.^1^, ^2^ After myocardial ischaemia, rapid restoration of blood flow is the most effective therapy to reduce the damage of myocardium, which brings oxygen and nutrition back. However, reperfusion can paradoxically cause the exacerbation of ischaemic tissue injury, which is called myocardial ischaemia/reperfusion (MI/R) injury,^3^, ^4^ and ultimately increases the overall size of myocardial infarct and leads to poor prognosis for patients with ischaemic heart disease.^5^ Myocardial cell apoptosis mainly occurs in the surviving portion of the heart which contributes to the loss of myocardial cells and inhibits left ventricular remodelling after MI/R injury, and the increase in myocardial cell apoptosis is positively correlated with the occurrence of cardiac failure after MI/R injury.^6^ So, apoptosis of myocardial cells is a key promoting factor contributing to cardiac failure after MI/R injury.^7^, ^8^ Therefore, inhibiting myocardial cell apoptosis is important for the prevention and treatment of MI/R injury.

Catechin is a bioactive polyphenol extracted from green tea that has antioxidant, antiviral and antioncogenic properties and is used to reduce the risk of various diseases.^9^, ^10^, ^11^ It has been well studied that EGCG, another bioactive polyphenol extracted from green tea, relieved MI/R injury through mitigating oxidative stress, decreasing apoptotic myocardial cells, reducing myocardial infarct size and ameliorating cardiac dysfunction in MI/R rabbit, mouse and rat models.^12^, ^13^ In hypoxia/reoxygenation (H/R)‐stimulated cell apoptosis of rat myocardial cells H9C2, EGCG inhibits the H/R‐treated cell apoptosis.^12^ Although catechin can protect rat heart from MI/R injury,^14^ the effect of catechin on H/R‐induced myocardial cell apoptosis is largely unknown.

Long non‐coding RNAs (lncRNAs) are a class of transcripts with a length longer than 200 nucleotides which can modulate the expressions of genes at epigenetic, translation or transcription level.^15^ More and more studies have shown that lncRNAs are involved in regulating myocardial cell apoptosis after MI/R injury.^16^, ^17^ Besides, lncRNA is involved in morphine‐meditated alleviation of autophage in MI/R injury,^18^ indicating that lncRNAs may mediate the protective role of drugs against MI/R injury. LncRNA myocardial infarction‐associated transcript (MIAT) is a widely expressed lncRNA that exerts its regulation function in cancers, diabetic retinopathy, atherosclerosis, etc^19^, ^20^, ^21^ Recent studies have shown that down‐regulation of lncRNA MIAT reduced myocardial cell apoptosis after MI/R injury, indicating targeting MIAT might protect against H/R‐induced myocardial cell apoptosis or MI/R injury.^22^, ^23^ Several studies have shown that the expression of lncRNA MIAT can be inhibited or promoted by drugs, indicating lncRNA MIAT involves in drug‐mediated inhibition or promotion of diseases progression.^21^, ^24^ However, whether lncRNA MIAT is involved in catechin‐mediated inhibition on MI/R injury is unclear.

In this study, we found that catechin down‐regulated lncRNA MIAT expression in myocardial tissue of MI/R rat and H/R‐induced myocardial cells. We further found that catechin relieved H/R‐induced myocardial cell apoptosis through regulating CREB/MIAT/Akt/Gsk‐3β pathway, which might clarify the underlying mechanism of catechin in inhibiting H/R‐induced myocardial cell apoptosis.

## 2 MATERIALS AND METHODS

### 2.1 Establishment of myocardial ischaemia/reperfusion (MI/R) rat model

Animal experiments were performed with the guidelines of the Animal Care and Use Committee of The Second Affiliated Hospital and Yuying Children's Hospital of Wenzhou Medical University. Male Sprague Dawley rats (7‐week‐old, 260‐300 g) were obtained from the laboratory animal centre of Wenzhou Medical University and maintained in a 12‐hour light/12‐hour dark room with free access to food and water for a week. Rats were divided into four groups, namely sham group, MI/R group, MI/R+Vehicle group and MI/R+Catechin group, with six rats in each group. Rats were intraperitoneally injected with 80 mg/kg sodium‐pentobarbital for anaesthesia. During the whole surgery, rats were placed in a controlled heating pad to keep the body temperature (37 ± 0.2°C) in the supine position, and electrocardiograph (ECG) was monitored in each rat. The establishment of MI/R rat model was conducted according to the previous report.^25^ Thoracotomy was performed at the fourth intercostal space, then the pericardium was cut, and a 5‐0 Prolene suture was placed around the left anterior descending coronary artery (LAD). The LAD was occluded for 30 minutes. To confirm the ischaemia was successfully developed by occlusion, prompt ST‐segment changes with progressive ST‐segment elevation in at least 3 leads with or without arrhythmia, and decolorization of occluded distal myocardium should be observed. Thirty minutes later, the ligation was released and reperfusion was continued for 24 hours. For rats in sham group, the procedure of surgery was the same as MI/R group, except the suture around the LAD was not tightened. For rats in MI/R+Vehicle group, 3 mL saline was intragastrically administrated into the rats for 10 days before surgery, and the procedure of surgery was the same as MI/R group. For rats in MI/R+Catechin group, 250 mg/kg/d catechin (C_15_H_14_O_6_; Sigma) was intragastrically administrated into the rats for 10 days before surgery, and the procedure of surgery was the same as MI/R group.

### 2.2 Electrocardiography

Serial echocardiograms were obtained at baseline (2 days before the occlusion of the LAD) and at 24 hours after surgery. PV loop conductance catheter (SPR‐869; Millar Instruments) was inserted into the left ventricular (LV) cavity through the right carotid artery. LV end‐systolic diameter (LVESd) and left ventricular end‐diastolic diameter (LVEDd) were calculated using the PVAN analysis software (Millar Instruments). Left ventricular ejection fraction (LVEF) = [(LVEDd^3^ − LVESd^3^)/LVEDd^3^] × 100%; Left ventricular fractional shortening (LVFS) = (LVEDd − LVESd)/LVEDd × 100%.^26^

### 2.3 Triphenyl‐tetrazolium‐chloride (TTC) staining and haematoxylin‐eosin (HE) staining

Prolene suture around the LAD was retightened, and Evans blue (1%) in PBS was retrogradely perfused into the aorta to visualize the area at risk. After the kill of rats, hearts were collected and frozen at −20°C. Heart tissues were sliced into 2‐mm‐thick sections, and 1% TTC was added to the sections and incubated at 37°C for 15 minutes. Intracellular dehydrogenases in the normal myocardium could react with TTC, whereas ischaemia area could not react with TTC and remained white. Heart sections stained with TTC were digitally scanned and evaluated using ImageJ software. Infarct size (%) was calculated as the ratio of infarct size/area at risk.

For HE staining, heart tissues were fixed with 4% paraformaldehyde at 25°C for 24 hours, embedded in paraffin, and sliced into 5‐μm‐thick sections. Then, Hematoxylin and Eosin Staining Kit (Beyotime Biotechnology) was used for staining according to the manufacturer's instructions.

### 2.4 Cell culture, treatment and transfection

Rat myocardial cell line H9C2 was obtained from the cell bank of Chinese Academy of Sciences. H9C2 cells were maintained in Dulbecco's Modified Eagle Medium (DMEM; Gibco) supplemented with 1.5 g/L NaHCO3, 10% foetal bovine serum (FBS; Gibco), 100 U/mL penicillin (Sinopharm Chemical Reagent) and 100 μg/mL streptomycin (Sinopharm Chemical Reagent) in a 5% CO_2_ incubator at 37°C.

For induction of hypoxia/reoxygenation (H/R), H9C2 cells were placed in a hypoxic chamber at 5% CO_2_, 95% N_2_ for 6 hours with serum/glucose‐free DMEM (Gibco). Subsequently, the cells were cultured in the glucose‐containing DMEM (Gibco) with 10% FBS under normoxic conditions for 12 hours to reoxygenation. Control cells were incubated under normoxic conditions. In H/R+Catechin groups, different concentrations of catechin (1, 5, 10, 20, 50 μmol/L) were added to the medium 0.5 hour before H/R induction. Catechin remained in the medium until the end of H/R treatment.

LY294002 (S1737) was from Beyotime Institute of Biotechnology (Shanghai, China). 10 μmol/L LY294002 was added to the medium 1 hour before H/R treatment.

Small interfering RNA (siRNA) oligonucleotides specific for MIAT (si‐MIAT) and cyclic adenosine monophosphate responsive element‐binding protein (CREB) (si‐CREB), MIAT overexpression vector (MIAT) and CREB overexpression vector (pcDNA‐CREB) and their negative controls (NC) were synthesized by RiboBio Co. Ltd. For cell transfection, H9C2 cells (2 × 10^5^) were seeded in 24‐well plates till 50% confluence, then transfected with MIAT overexpression vector, si‐MIAT, si‐CREB, pcDNA‐CREB, and their negative controls using Lipofectamine 2000 (Invitrogen).

### 2.5 Quantitative real‐time polymerase chain reaction (qRT‐PCR)

Total RNAs from myocardium or H9C2 cells were extracted using TRIzol Reagent (Invitrogen), and then, RNA was reverse‐transcribed to cDNA using High Capacity cDNA Reverse Transcription Kit (Applied Biosystems). qRT‐PCR was performed in a QuantStudio 5 Real‐Time PCR System (Applied Biosystems) using SuperScript IV One‐Step RT‐PCR System (Invitrogen). Each sample was quantified in a volume of 20 μL containing cDNA (1 μL). The relative lncRNA expressions of LncRNA MIAT, lncRNA ROR, lncRNA HRIM, lncRNA MALAT1, lncRNA UCA1 and lncRNA NRF were normalized to GAPDH and calculated using 2^−ΔΔct^ method.

### 2.6 Cell counting kit‐8 (CCK‐8) assay

H9C2 cells (5 × 10^3^/well) were seeded into 96‐well plates with different treatments. CCK‐8 solution (10 μL; Beyotime Biotechnology) was added into each well and incubated at 37°C for 1 hour. Cell viability normalized to cells without treatment was measured when the absorbance at 450 nm using iMark Microplate Absorbance Reader (Bio‐Rad).

### 2.7 Flow cytometry

eBioscience Annexin V‐FITC Apoptosis Detection Kit (Invitrogen) was used to detect the apoptosis of H9C2 cells. H9C2 cells were collected and washed with PBS for twice by gentle shaking. Then, 200 μL 1× binding buffer was used to re‐suspend H9C2 cells (3 × 10^5^/mL). For 195 μL cell suspension, 5 μL Annexin V‐FITC was added and incubated at 25°C for 10 minutes. 1× binding buffer (200 μL) was used to wash cells again. For 195 μL cell suspension, 10 μL propidium iodide was added and then flow cytometry analysis of stained cells was performed using a FACSCanto II flow cytometry (BD Biosciences).

Intracellular reactive oxygen species (ROS) production was detected using Reactive Oxygen Species Assay Kit (Beyotime Biotechnology).^27^ DCFH‐DA diffused easily across the cell membrane and hydrolysed by intracellular esterase to form non‐fluorescent DCFH. If ROS was present, non‐fluorescent DCFH was oxidized rapidly to form high fluorescence DCF. DCFH‐DA (10 μmol/L) was added to cells and incubated at 37°C for 20 minutes. Relative fluorescent intensity was obtained using flow cytometry with excitation wavelength at 488 nm and emission wavelengths at 525 nm.

### 2.8 Dual‐luciferase reporter assay

Dual‐luciferase reporter assay was used to detect the interaction between CREB and MIAT promoter region. The wild‐type MIAT promoter luciferase (Luc) and mutant MIAT promoter luciferase were constructed by Genomeditech Co. Ltd. HEK293T cells were seeded in 24‐well plates, and then, luciferase reporter constructs (300 ng/well) were cotransfected into HEK293T cells with 600 ng/well pcDNA‐NC and pcDNA‐CREB using Lipofectamine 2000 (Invitrogen). Forty‐eight hours later, Dual‐Luciferase Reporter Assay kit (Promega) was used to measure the relative luciferase activity.

### 2.9 Detection of mitochondrial membrane potential (MMP)

MMP was detected using JC‐10 Mitochondrial Membrane Potential Assay Kit (Abcam). H9C2 cells were collected, washed with PBS and seeded into 96‐well plates. Then, cells were incubated with JC‐10 dye‐loading solution (50 µL/well) and incubated at 37°C for 20 minutes. Assay buffer B (50 µL/well) was added to each well. The fluorescence intensity of stained cells was subjected to a fluorescence microplate reader (Bio‐Rad), and the value of MMP was expressed as ratios of fluorescence intensity at 590/525 nm.

### 2.10 Chromatin Immunoprecipitation (ChIP) assay

ChIP assay was conducted using Pierce™ Agarose ChIP Kit (Thermo Fisher Scientific) according to the manufacturer's instructions. Chromatin was extracted, and DNA was cut to 0.2‐kb to 1‐kb fragments. H9C2 cells were cross‐linked using 1% formaldehyde, and the chromatin was immunoprecipitated with the antibody CREB (Abcam). IgG was used as a negative control, and H3 was used as a positive control. DNA was re‐suspended in 50 μL of TE buffer and amplified by PCR. PCR products were determined on a 1.5% agarose gel. ChIP‐PCR primers used in this study as follows: CREB site 2, F: 5′‐CCATGCCTGACTGCACTC‐3′, R: 5′‐AAGATACGAGCCATAAGATCACA‐3′; CREB site 4, F: 5′‐TGGAAGCATGAGTTCAAATCT‐3′, R: 5′‐ACCTGTAGGACTCGGAAGAC‐3′.

### 2.11 Western blot

Total proteins were isolated from H9C2 cells using RIPA buffer (Beyotime Biotechnology) containing phenylmethanesulfonyl fluoride (PMSF; Beyotime Biotechnology). The concentration of protein samples was detected by Pierce BCA Protein Assay Kit (Thermo Fisher Scientific). Protein samples were separated by sodium dodecyl‐sulphate polyacrylamide gel electrophoresis and transferred to polyvinylidene fluoride membranes (Invitrogen). Then, membranes were blocked in 5% skim milk for 1 hour and incubated with primary antibodies CREB (1:500 dilution; Abcam), Bax (1:1000 dilution; Abcam), Bcl‐2 (1:2000 dilution; Abcam), cleaved caspase‐3 (1:500 dilution; Abcam), and cleaved caspase‐9 (1:1000 dilution; Cell Signaling Technology) at 4°C overnight. The membranes were the incubated with second antibodies goat‐anti‐mouse horseradish peroxidase (HRP)‐conjugated antibody (1:2000 dilution; Abcam) and goat‐anti‐rabbit HRP‐conjugated antibody (1:2000 dilution; Abcam) at 25°C for 1 hour. The intensity of bands was analysed using ChemiDoc MP imaging system (BIO‐RAD).

### 2.12 Statistical analysis

Statistics were calculated using SPSS 17.0 (Chicago, IL, USA), and all data were presented as mean ± standard deviation (SD). Statistical analysis between groups or among groups was analysed using Student's *t* test or one‐way ANOVA followed by Bonferroni post hoc test. *P* value < .05 was considered statistically significant.

## 3 RESULTS

### 3.1 Catechin improved heart function of myocardial ischaemia/reperfusion (MI/R) rat and down‐regulated lncRNA MIAT expression in myocardial tissue

**Figure 1:**
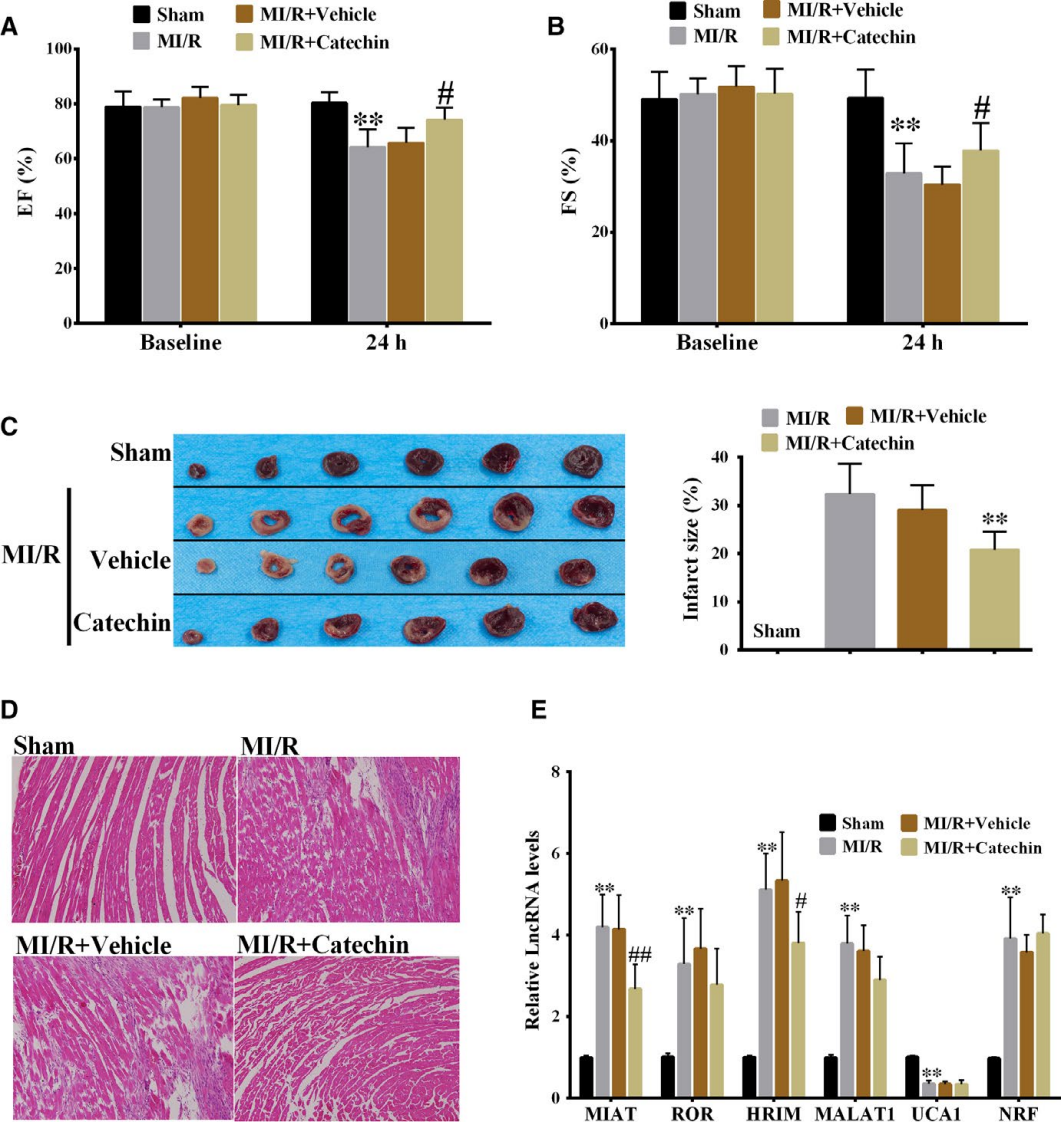
Catechin improved heart function of myocardial ischaemia/reperfusion (MI/R) rat and down‐regulated lncRNA MIAT expression in myocardial tissue. SD rats were divided into Sham group, MI/R group, MI/R+Vehicle group and MI/R+Catechin group, with six rats in each group. Echocardiography was used to detect heart function of rats, and the data of left ventricular end‐systolic diameter (LVESd) and left ventricular end‐diastolic diameter (LVEDd) were obtained. A, Left ventricular ejection fraction (LVEF). B, Left ventricular fractional shortening (LVFS). LVEF = [(LVEDd^3^ − LVESd^3^)/LVEDd^3^] × 100%; LVFS = (LVEDd − LVESd)/LVEDd × 100%. **P < .01 vs Sham; #*P* < .05 vs MI/R+Vehicle. C, TTC staining of myocardial tissue. **P < .01 vs MI/R+Vehicle. D, HE staining of myocardial tissue. Magnification ×200. E, LncRNA MIAT, lncRNA ROR, lncRNA HRIM, lncRNA MALAT1, lncRNA UCA1 and lncRNA NRF expressions in myocardial tissue were detected using qRT‐PCR. **P < .01 vs Sham; ##*P* < .01, #*P* < .05 vs MI/R+Vehicle. N = 6

According to the analysis of data from echocardiography, we found catechin significantly increased left ventricular ejection fraction (LVEF) and left ventricular fractional shortening (LVFS) in MI/R+Catechin group than MI/R+Vehicle group (Figure 1A,B), indicating that catechin improved heart function of MI/R rat. TTC staining showed that catechin significantly decreased infract size in MI/R+Catechin group than MI/R+Vehicle group (Figure 1C). HE staining showed myocardial fibrinolysis and inflammatory cell infiltration in MI/R rat. Compared with MI/R group and MI/R+Vehicle group, better myocardial fibre structure and less inflammatory cell infiltration were observed in MI/R+Catechin group (Figure 1D). These findings indicated that catechin relieved myocardial injury. Previous reports have shown that LncRNA MIAT, lncRNA ROR, lncRNA HRIM, lncRNA MALAT1, lncRNA UCA1 and lncRNA NRF were involved in the regulation of MI/R,^22^, ^28^, ^29^ so we detected the expressions of these lncRNAs and selected lncRNAs that might be regulated by catechin. As shown in Figure 1E, catechin significantly decreased lncRNA MIAT and lncRNA HRIM expressions in myocardial tissue, and catechin had a more significant inhibitory effect on lncRNA MIAT. Therefore, we will further investigate whether lncRNA MIAT is involved in the relief of myocardial injury mediated by catechin.

### 3.2 Catechin relieved hypoxia/reoxygenation (H/R)‐induced myocardial cell apoptosis, and lncRNA MIAT overexpression attenuated the protective effect of catechin on myocardial cells

**Figure 2:**
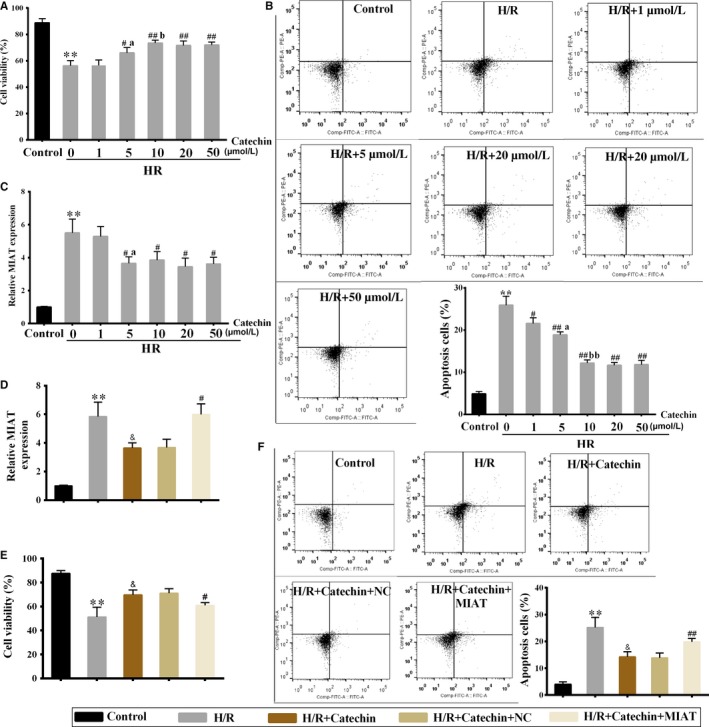
Catechin relieved hypoxia/reoxygenation (H/R)‐induced myocardial cell apoptosis, and lncRNA MIAT overexpression attenuated the protective effect of catechin on myocardial cells. Rat myocardial cells H9C2 were divided into control group, H/R group and H/R+ (1, 5, 10, 20, 50 μmol/L) Catechin groups. Catechin was added to the medium 0.5 h before H/R induction and remained in the medium until the end of H/R treatment. A, Cell viability of H9C2 cells was detected using CCK‐8 assay. B, The apoptosis of H9C2 cells was detected by flow cytometry. C, MIAT expression was detected by qRT‐PCR. **P < .01 vs control; ##*P* < .01, #*P* < .05 vs H/R; ^a^ *P* < .05 vs H/R+1 μmol/L catechin; ^b^ *P* < .05, ^bb^ *P* < .01 vs H/R+5 μmol/L catechin. Then, H9C2 cells were divided into control group, H/R group, H/R+Catechin group, H/R+Catechin+NC group and H/R+Catechin+MIAT (MIAT overexpressing vector) group. D, MIAT expression in H9C2 cells was detected by qRT‐PCR. E, Cell viability of H9C2 cells was detected using CCK‐8 assay. F, The apoptosis of H9C2 cells was detected by flow cytometry. **P < .01 vs control; &*P* < .05 vs H/R; #*P* < .05, ##*P* < .01 vs H/R+Catechin+NC. N = 3

Firstly, we found that there were no significant effect of catechin on cell viability and apoptosis of H9C2 cells (Figure S1). To observe the effect of catechin on cell viability and apoptosis of H9C2 cells under H/R condition, catechin was added to the medium 0.5 hour before H/R induction. As shown in Figure 2A, catechin (≥5 μmol/L) significantly increased cell viability under H/R condition. Catechin (≥1 μmol/L) significantly reduced the apoptosis of H9C2 cells under H/R condition (Figure 2B). In addition, H/R treatment significantly increased MIAT expression in H9C2 cells, and catechin (≥5 μmol/L) significantly inhibited H/R‐induced up‐regulation of MIAT (Figure 2C).

Next, we further investigated whether MIAT was involved in protection of myocardial cell injury mediated by catechin. As described above, compared with the control group, MIAT was obviously increased (Figure 2D), cell activity was obviously decreased (Figure 2E) and apoptosis was obviously increased in the H/R group (Figure 2F). However, catechin (10 μmol/L) can reverse the H/R effects. LncRNA MIAT expression was significantly up‐regulated in H9C2 cells from H/R+Catechin+MIAT group, indicating MIAT overexpressing vector could effectively increase MIAT expression in H9C2 cells (Figure 2D). Cell viability was also inhibited in H/R+Catechin+MIAT group than H/R+Catechin+NC group (Figure 2E), and the apoptosis of H9C2 cells was promoted in H/R+Catechin+MIAT group than H/R+Catechin+NC group (Figure 2F), indicating lncRNA MIAT overexpression attenuated the protective effect of catechin on myocardial cells.

### 3.3 CREB inhibited lncRNA MIAT expression at transcriptional level and catechin suppressed lncRNA MIAT expression through up‐regulating CREB

**Figure 3:**
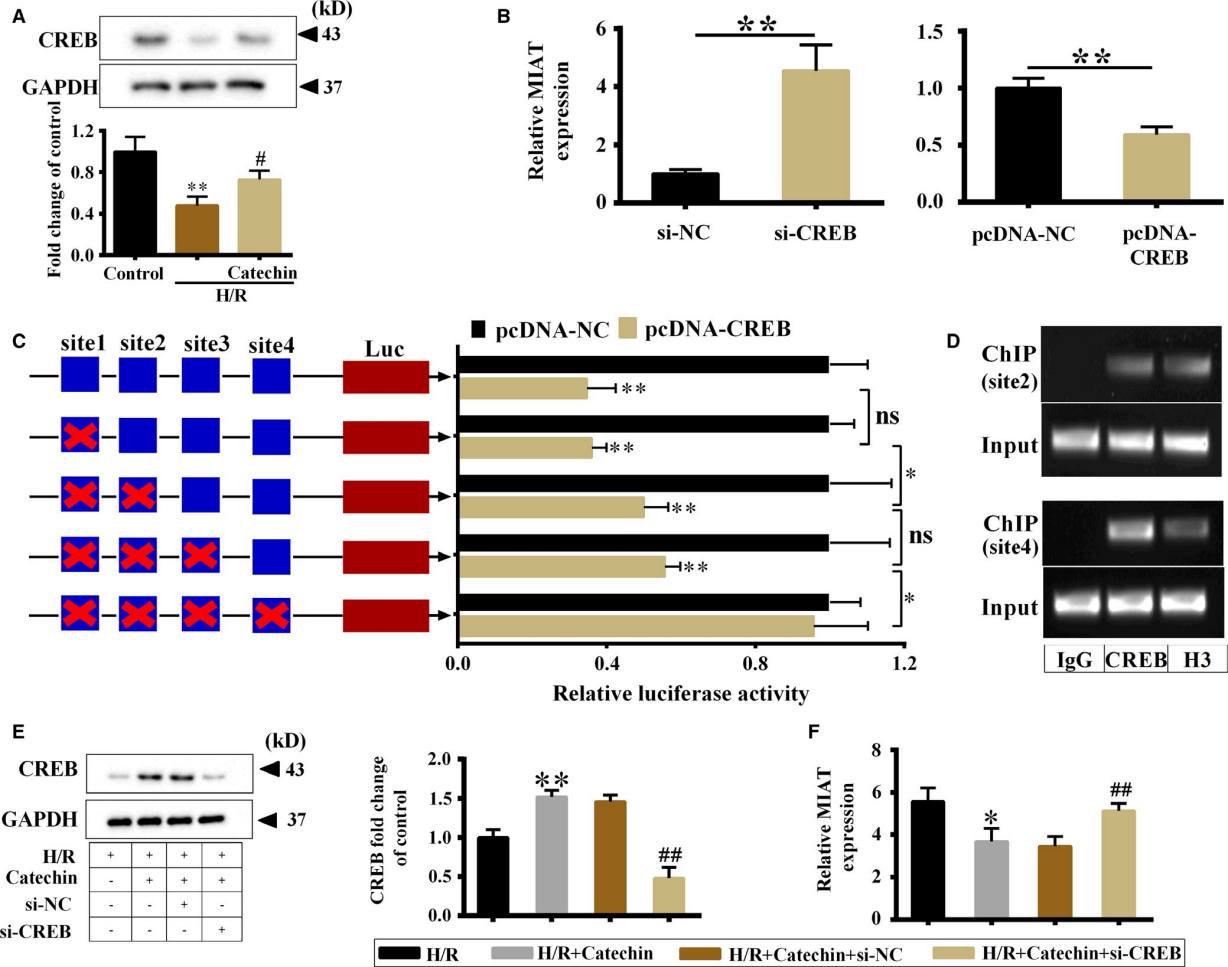
Catechin suppressed lncRNA MIAT expression through up‐regulating CREB. A, H9C2 cells were divided into control group, H/R group and H/R+Catechin group. CREB protein level was detected by Western blot. **P < .01 vs control; #*P* < .05 vs H/R. B, H9C2 cells were divided into si‐NC, si‐CREB, pcDNA‐NC and pcDNA‐CREB groups. qRT‐PCR was used to detect MIAT expression. **P < .01 vs si‐NC or pcDNA‐NC. C, Wild‐type MIAT promoter luciferase (Luc) and mutant MIAT promoter luciferase were constructed, and they cotransfected into HEK293T cells with pcDNA‐NC and pcDNA‐CREB. The luciferase activity was detected by dual‐luciferase reporter gene assay. *P < .05; **P < .01 vs pcDNA‐NC. D, CREB occupancy on the MIAT promoter was detected by ChIP assay. IgG was used as negative control, H3 was used as positive control. H9C2 cells were divided into H/R group, H/R+Catechin group, H/R+Catechin+si‐NC group and H/R+Catechin+si‐CREB group. E, CREB protein level was detected by Western blot. F, MIAT expression was detected by qRT‐PCR. **P < .01, *P < .05 vs H/R; ##*P* < .01 vs H/R+Catechin+si‐NC. N = 3

Previous reports have shown that the transcription of lncRNAs can be regulated by transcription factors at the transcriptional level under physiological and pathological conditions,^30^, ^31^ so we further explore the possible mechanism of catechin in the regulation of MIAT expression. According to the prediction of bioinformatics software JASPAR, several transcription factors could bind to the MIAT promoter region to regulate MIAT expression. Transcription factor CREB is one of these transcription factors and has been proved to be involved in the regulation of hypoxia‐induced apoptosis of myocardial cells.^32^ So, we firstly detected whether catechin changed CREB protein level under H/R condition. Western blot showed that H/R treatment significantly down‐regulated CREB protein level, and catechin significantly up‐regulated CREB protein level under H/R condition (Figure 3A). As shown in Figure S2, knockdown or overexpress CREB by cell transfection with si‐CREB and si‐NC (control) or pcDNA‐CREB and pcDNA‐NC (control). We found that CREB knockdown significantly increased MIAT expression, and CREB overexpression significantly decreased MIAT expression (Figure 3B), indicating that CREB negatively regulated MIAT expression.

We noticed there were four potential binding sites for CREB in the MIAT promoter region (binding site 1: −1765 to −1758, binding site 2: −1420 to −1413, binding site 3: −1209 to −1202, binding site 4: −1009 to −1002) according to the prediction of JASPAR. Next, the interaction of CREB with four potential binding sites on the MIAT promoter was detected in HEK293T cells. The luciferase activity was significantly decreased in pcDNA‐CREB group (Figure 3C), indicating that CREB could interact with MIAT promoter. The luciferase activity was significantly increased after mutation of binding sites 1 + 2 than mutation of binding site 1, and the luciferase activity was significantly increased after mutation of binding sites 1 + 2 + 3 + 4 than mutation of binding sites 1 + 2 + 3, indicating binding sites 2 and 4 play important roles in the interaction of CREB with MIAT promoter region (Figure 3C). CREB occupancy on the MIAT promoter was detected by ChIP (Figure 3D), indicating that CREB could bind to binding sites 2 and 4. We divided H9C2 cells into four groups: H/R group, H/R+Catechin group, H/R+Catechin+si‐NC and H/R+Catechin+si‐CREB group. We found that the CREB protein was significantly increased and the MIAT was significantly decreased in the H/R+Catechin group compared with the H/R group (Figure 3D,E). However, after transfection of si‐CREB, CREB protein was significantly reduced while MIAT was significantly elevated (Figure 3D,E).

### 3.4 Catechin increased myocardial mitochondrial membrane potential (MMP), decreased intracellular ROS level and cytochrome c level under H/R condition, and lncRNA MIAT overexpression abolished the effects of catechin

**Figure 4:**
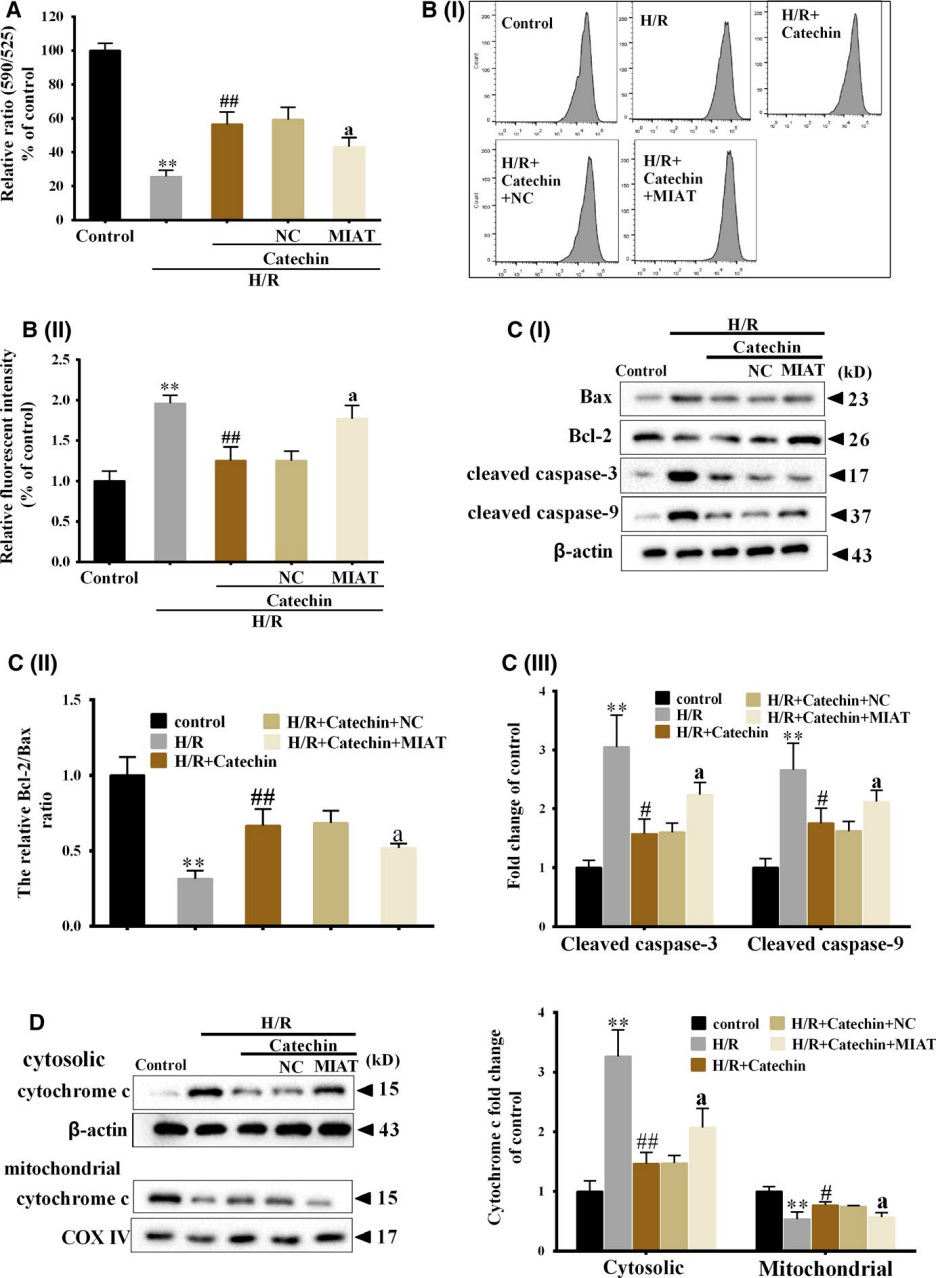
Catechin increased myocardial mitochondrial membrane potential, decreased intracellular ROS level and cytochrome c level under H/R condition, and lncRNA MIAT overexpression abolished the effects of catechin. H9C2 cells were divided into control, H/R, H/R+Catechin, H/R+Catechin+NC and H/R+Catechin+MIAT groups. H9C2 cells were transfected with NC or MIAT overexpressing vector, and then catechin and H/R treatment were conducted. A, Mitochondrial membrane potential of H9C2 cells was detected using JC‐10 Mitochondrial Membrane Potential Assay Kit. B, Intracellular ROS level was detected by flow cytometry. C, Protein levels of Bax, Bcl‐2, cleaved caspase‐3 and cleaved caspase‐9 were detected by Western blot. D, Cytosolic and mitochondrial cytochrome c protein level was detected by Western blot. **P < .01 vs control; #*P* < .05, ##*P* < .01 vs H/R; ^a^ *P* < .05 vs H/R+Catechin+NC. N = 3

As shown in Figure 4A, H/R treatment significantly decreased MMP in H9C2 cells, catechin significantly increased MMP in H9C2 cells, and MIAT overexpression further decreased MMP in H9C2 cells. Besides, H/R treatment significantly promoted intracellular ROS level, catechin significantly decreased intracellular ROS level, and MIAT overexpression further increased intracellular ROS level in H9C2 cells (Figure 4B). Protein levels of Bax, Bcl‐2, cleaved caspase‐3, cleaved caspase‐9, cytosolic and mitochondrial cytochrome c were also detected, and results showed that catechin increased Bcl‐2/Bax ratio and decreased cleaved caspase‐3, cleaved caspase‐9 and cytochrome c protein levels under H/R condition, whereas MIAT overexpression abolished the effects of catechin (Figure 4C,D).

### 3.5 Catechin improved mitochondrial function and relieved apoptosis of myocardial cells through promoting Akt/Gsk‐3β activation under H/R condition

**Figure 5:**
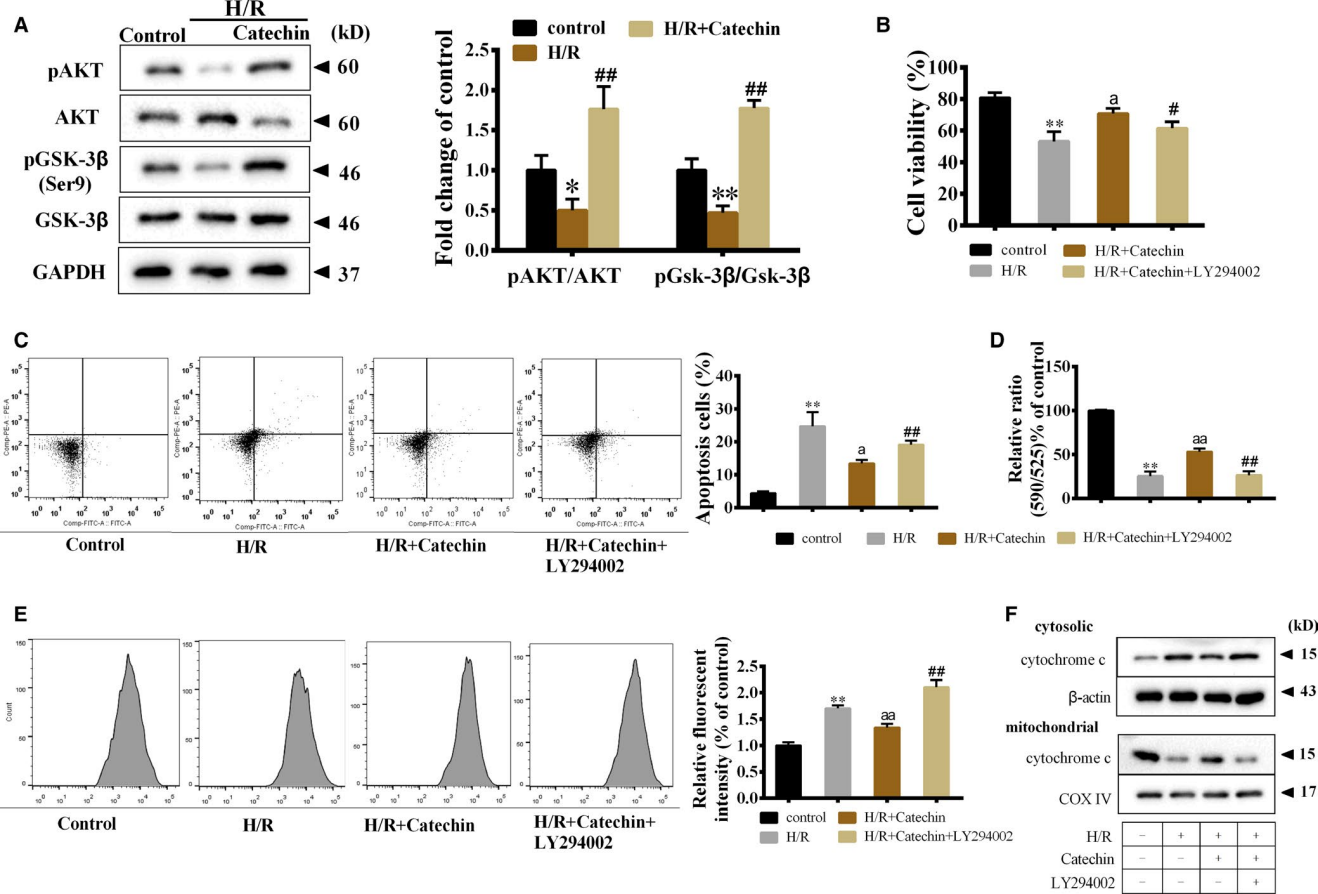
Catechin improved mitochondrial function and relieved apoptosis of myocardial cells through promoting Akt/Gsk‐3β activation under H/R condition. A, H9C2 cells were divided into control, H/R and H/R+Catechin groups. Protein levels of AKT, pAKT, Gsk‐3β and pGsk‐3β were detected using Western blot. *P < .05, **P < .01 vs control; ##*P* < .01 vs H/R. H9C2 cells were divided into control group, H/R group, H/R+Catechin group and H/R+Catechin+10 μmol/L LY294002 group (a potent PI3K inhibitor, which was added into the medium 1 h before H/R induction). B, Cell viability of H9C2 cells was detected by CCK‐8 assay. C, The apoptosis of H9C2 cells was detected by flow cytometry. D, Mitochondrial membrane potential of H9C2 cells was detected using JC‐10 Mitochondrial Membrane Potential Assay Kit. E, Intracellular ROS level was detected by flow cytometry. **P < .01 vs control; ^a^ *P* < .05, ^aa^ *P* < .01 vs H/R; ##*P* < .01 vs H/R+Catechin. F, Cytosolic and mitochondrial cytochrome c protein level was detected by Western blot. N = 3

Under H/R condition, catechin significantly increased pAKT/AKT ratio and pGsk‐3β/Gsk‐3β ratio (Figure 5A), indicating catechin promoted Akt/Gsk‐3β activation. To observe whether catechin exerted its protective role in myocardial cells through promoting Akt/Gsk‐3β activation, LY294002 (a potent PI3K inhibitor) was used to treat H9C2 cells. We found that under H/R and catechin treatment, LY294002 significantly inhibited cell viability of H9C2 cells (Figure 5B), promoted the apoptosis of H9C2 cells (Figure 5C), inhibited MMP in H9C2 cells (Figure 5D), increased intracellular ROS level (Figure 5E) and up‐regulated cytosolic and mitochondrial cytochrome c protein levels (Figure 5F). These findings indicated that under H/R condition, catechin improved mitochondrial function and reduced apoptosis of H9C2 cells through promoting Akt/Gsk‐3β activation.

### 3.6 MIAT inhibited Akt/Gsk‐3β activation and promoted the apoptosis of myocardial cells under H/R condition

**Figure 6:**
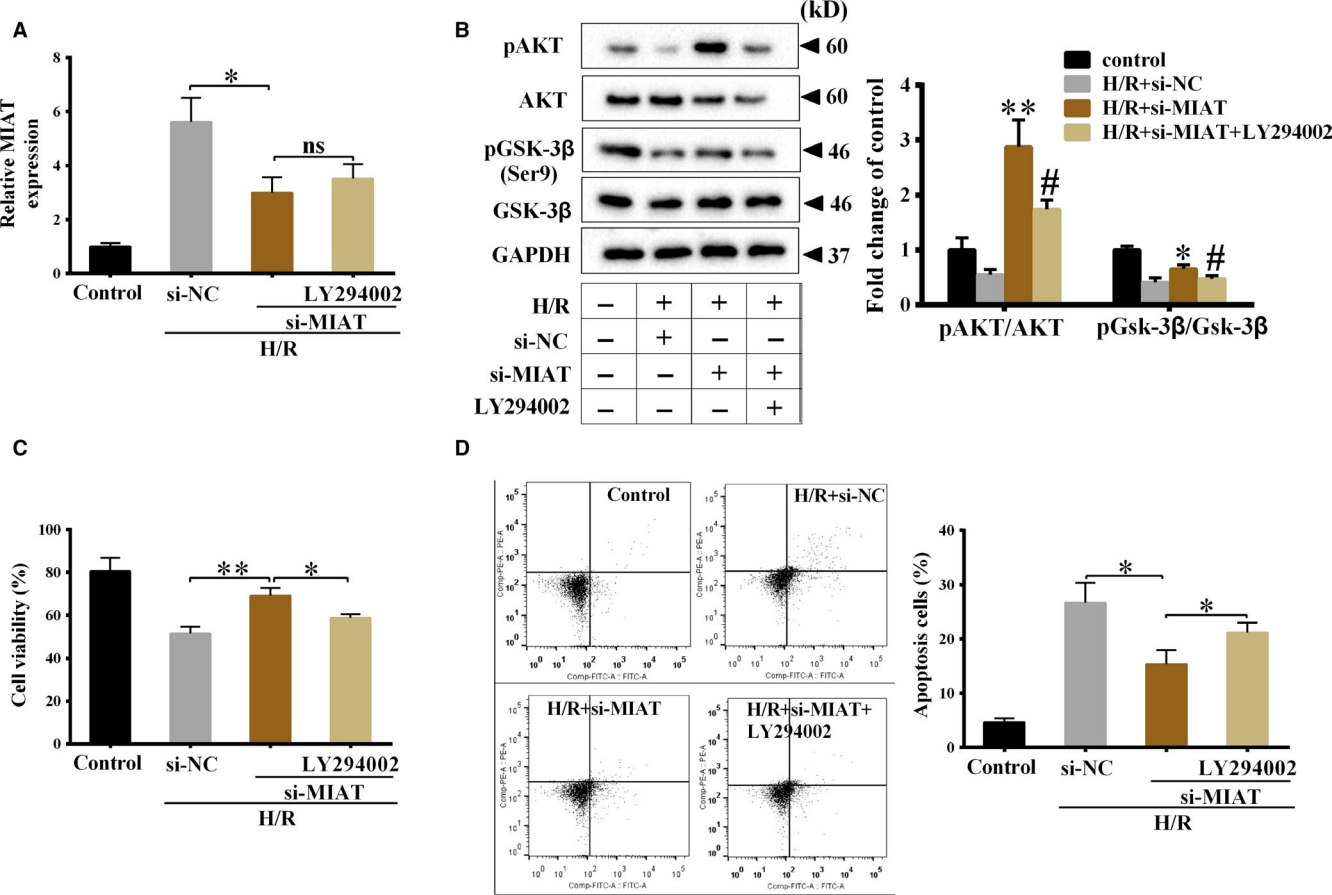
MIAT inhibited Akt/Gsk‐3β activation and promoted the apoptosis of myocardial cells under H/R condition. H9C2 cells were divided into control, H/R+si‐NC, H/R+si‐MIAT and H/R+si‐MIAT+LY294002 groups. A, MIAT expression was detected by qRT‐PCR. *P < .05, **P < .01. B, Protein levels of AKT, pAKT, Gsk‐3β and pGsk‐3β were detected using Western blot. *P < .05, **P < .01 vs H/R+si‐NC; #*P* < .05 vs H/R+si‐MIAT. C, Cell viability of H9C2 cells was detected by CCK‐8 assay. *P < .05, **P < .01. D, The apoptosis of H9C2 cells was detected by flow cytometry. *P < .05, **P < .01. N = 3

As shown in Figure 6A, si‐MIAT significantly down‐regulated MIAT expression under H/R condition, whereas LY294002 did not significantly change MIAT expression under H/R condition, indicating Akt/Gsk‐3β pathway did not regulate MIAT expression. Under H/R condition, si‐MIAT significantly increased pAKT/AKT ratio and pGsk‐3β/Gsk‐3β ratio, indicating MIAT inhibited Akt/Gsk‐3β activation (Figure 6B). LY294002 further decreased pAKT/AKT ratio and pGsk‐3β/Gsk‐3β ratio (Figure 6B). Besides, si‐MIAT significantly promoted cell viability of H9C2 cells and reduced the apoptosis of H9C2 cells under H/R condition, LY294002 further inhibited cell viability and increased the apoptosis of H9C2 cells (Figure 6C,D). These findings suggested that under H/R condition, MIAT inhibited cell viability and promoted the apoptosis of H9C2 cells through inhibiting Akt/Gsk‐3β activation.

### 3.7 Catechin promoted Akt/Gsk‐3β activation through inhibiting MIAT expression under H/R condition

**Figure 7:**
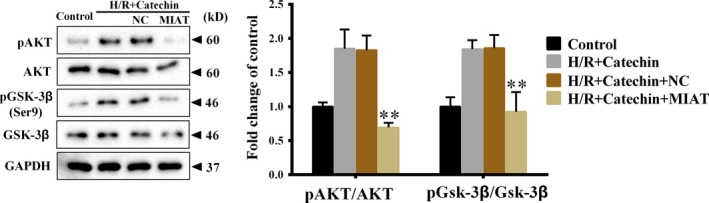
Catechin promoted Akt/Gsk‐3β activation through inhibiting MIAT expression under H/R condition. H9C2 cells were divided into control group, H/R+Catechin group, H/R+Catechin+NC group and H/R+Catechin+MIAT group. Protein levels of AKT, pAKT, Gsk‐3β and pGsk‐3β were detected using Western blot. **P < .01 vs H/R+Catechin+NC. N = 3

As shown in Figure 7, catechin significantly increased pAKT/AKT ratio and pGsk‐3β/Gsk‐3β ratio under H/R condition, whereas MIAT overexpression significantly decreased pAKT/AKT ratio and pGsk‐3β/Gsk‐3β ratio, indicating that under H/R condition, catechin promoted Akt/Gsk‐3β activation through inhibiting MIAT expression.

## 4 DISCUSSION

LncRNAs are the cellular responding molecules for bioactive polyphenol treatment in various diseases. For example, studies showed that lncRNAs are dysregulated after EGCG treatment in lung cancer, nasopharyngeal carcinoma, prostatic hyperplasia, osteoarthritis, etc^33^, ^34^, ^35^ However, whether lncRNAs mediated the protective role of catechin, a kind of bioactive polyphenol that has a variety of pharmacological and biological properties and plays a protective role in MI/R injury,^14^ against H/R‐induced myocardial cell apoptosis or MI/R injury is largely unknown. In this study, we found that lncRNA MIAT expression was down‐regulated after catechin treatment in myocardial tissue of MI/R rat and H/R‐induced myocardial cells, indicating lncRNA MIAT might be involved in the protective role of catechin against MI/R injury or H/R‐induced myocardial cell apoptosis. Further in vitro experiments showed catechin relieved H/R‐induced myocardial cell apoptosis, and lncRNA MIAT overexpression attenuated the protective effect of catechin on myocardial cells, indicating catechin relieved H/R‐induced myocardial cell apoptosis via down‐regulating lncRNA MIAT, which provided evidence that catechin could regulate lncRNA to relieve H/R‐induced myocardial cell apoptosis.

Same as the transcriptional regulation of other genes, mounting evidence showed that the transcription of lncRNAs could be regulated by transcription factors at the transcriptional level under physiological and pathological conditions.^36^, ^37^ Researchers have found that lncRNA MIAT can be regulated by transcription factor Oct4 in the differentiation of mouse embryonic stem cells.^38^ LncRNA MIAT can also be positively regulated by transcription factor c‐Myc to promote Müller cell‐derived proinflammatory cytokine release.^21^ These findings indicated that lncRNA MIAT can be transcriptionally regulated by transcription factors. So, whether the transcription of lncRNA MIAT was regulated by transcription factors in H/R‐induced myocardial cells was analysed in this study. Basing on the prediction of bioinformatics software JASPAR, we got several transcription factors that have potential ability to bind to the MIAT promoter region. Among these transcription factors, CREB, a transcription factor that was involved in the regulation of hypoxia‐induced myocardial cell apoptosis,^32^ attracted our attention. Our data showed that CREB expression was significantly down‐regulated after H/R treatment, while this down‐regulation was inhibited by catechin treatment in H/R‐induced H9C2 cells, indicating CREB might be involved in the protection of catechin in H/R‐induced myocardial cell injury. Therefore, we investigated whether CREB acted as a transcription factor to regulate lncRNA MIAT expression in H/R‐induced myocardial cells. By loss‐ and gain‐of‐function experiments, we found that CREB negatively regulated MIAT expression in H/R‐induced H9C2 cells. Then, the results of dual‐luciferase reporter gene assay showed that CREB could bind to the MIAT promoter region. Moreover, under Catechin+H/R treatment, silencing CREB promoted MIAT expression in H9C2 cells. Therefore, we clarified that lncRNA MIAT can be regulated by CREB in H/R‐induced myocardial cells for the first time.

Recently, studies have shown that abnormally expressed lncRNAs were closely related with mitochondrial dysfunction.^39^ For example, lncRNA HOTAIR knockdown increased ROS production and caused mitochondrial swelling in HeLa cells, indicating lncRNA HOTAIR knockdown led to mitochondrial dysfunction.^40^ Overexpression of lncRNA UIHTC enhanced mitochondrial function to protect against myocardial infarction.^41^ These findings indicated lncRNAs exerted a critical role in mitochondrial dysfunction. It has been proved that during MI/R injury, mitochondria produced excessive ROS, which further induced mitochondrial permeability transition pore opening, resulting mitochondrial function reduction and myocardial cell apoptosis.^42^, ^43^ Importantly, catechin could reduce cell apoptosis through maintaining mitochondrial function.^44^ Therefore, catechin may improve mitochondrial function to reduce myocardial cell apoptosis through regulating lncRNA. In this study, we found catechin increased myocardial MMP and decreased intracellular ROS level, cytochrome c level and apoptosis‐related proteins levels in H/R‐induced H9C2 cells, and lncRNA MIAT overexpression abolished the effects of catechin, indicating catechin improved mitochondrial function to reduce H9C2 cell apoptosis through down‐regulating lncRNA MIAT expression.

Akt/Gsk‐3β pathway has been shown to be involved in modulating oxidative stress and apoptosis, and the activation of Akt/Gsk‐3β pathway preserved heart function and alleviated MI/R injury.^45^, ^46^ Activation of Akt/Gsk‐3β pathway also inhibited the apoptosis and oxidative stress of H/R‐induced H9C2 cells.^47^, ^48^ Importantly, the activation of Akt/Gsk‐3β pathway is involved in the cardioprotection function of EGCG.^49^ In this study, we clarified that the Akt/Gsk‐3β pathway was activated by catechin and this activation was mediated by MIAT down‐regulation in H/R‐induced H9C2 cells. To the best of our knowledge, no other studies figured out catechin improved mitochondrial function and reduced the apoptosis of H/R‐induced H9C2 cells through regulating lncRNA MIAT/Akt/Gsk‐3β pathway, which revealed the underlying mechanism of catechin in protecting against H/R‐induced myocardial cell apoptosis.

In conclusion, this study showed that catechin relieved H/R‐induced myocardial cell apoptosis through regulating CREB/lncRNA MIAT/Akt/Gsk‐3β pathway, which clarified the underlying mechanism of catechin in inhibiting H/R‐induced myocardial cell apoptosis, and might provide potential targets for relieving MI/R injury.

## CONFLICT OF INTEREST

The authors declare no conflict of interests.

## AUTHOR CONTRIBUTIONS

Mingyuan Huang contributed to the conception of the study; Lin Cong performed the experiments, analysed the data and wrote the paper; Yisheng Su and Dazhen Wei participated in the execution of the experiment; Lu Qian, Dawei Xing and Jialin Pan collected the literature. Mingyuan Huang and Ye Chen corrected the manuscript.

## Data Availability

For some reasons, the findings of this study are available do not support availability.
