# Supplementary material for: Catechin relieves hypoxia/reoxygenation‐induced myocardial cell apoptosis via down‐regulating lncRNA MIAT
Source: J Cell Mol Med. 2020 Jan 19;24(3):2356–68. doi: 10.1111/jcmm.14919 (PMC7011153; doi:10.1111/jcmm.14919)
Supplement: Supplementary file 3 [file JCMM-24-2356-s003.docx]

Supplement Figure S1. Effect of Catechin on myocardial cell viability and apoptosis. H9C2 cells were divided into control group and Catechin groups (1, 5, 10, 20, 50 μM). A. CCK-8 assay was used to detect cell viability. B. Flow cytometry was used to detect cell apoptosis. N=3.

Supplement Figure S2. CREB protein level was promoted in H9C2 cells transfected with pcDNA-CREB. **p<0.01 vs si-NC, ##p<0.01 vs pcDNA-NC. N=3.
